# Possible Association of Periodontal Diseases With *Helicobacter pylori* Gastric Infection: A Systematic Review and Meta-Analysis

**DOI:** 10.3389/fmed.2022.822194

**Published:** 2022-04-19

**Authors:** Nansi López-Valverde, Bruno Macedo de Sousa, Antonio López-Valverde, Ana Suárez, Cinthia Rodríguez, Juan Manuel Aragoneses

**Affiliations:** ^1^Department of Surgery, University of Salamanca, Salamanca, Spain; ^2^Instituto de Investigación Biomédica de Salamanca (IBSAL), Salamanca, Spain; ^3^Institute for Occlusion and Orofacial Pain Faculty of Medicine, University of Coimbra, Coimbra, Portugal; ^4^Department of Preclinical Dentistry, School of Biomedical Sciences, Universidad Europea de Madrid, Madrid, Spain; ^5^Department of Dentistry, Universidad Federico Henríquez y Carvajal, Santo Domingo, Dominican; ^6^Faculty of Dentistry, Universidad Alfonso X El Sabio, Madrid, Spain

**Keywords:** *Helicobacter pylori*, dental plaque, periodontal diseases, gastric infection, saliva

## Abstract

Some research has suggested that dental plaque and saliva could be reservoirs of *Helicobacter pylori* (*H. pylori*) and be capable of infecting or re-infecting the gastric mucosa after eradication, with certain studies showing a significant association between PD and gastric infection by this bacterium. An electronic search was performed in PubMed, EMBASE, and Web of Science databases with the terms “*Helicobacter pylori* AND periodontal diseases”; “*Helicobacter pylori* AND gingivitis”; “*Helicobacter pylori* AND chronic periodontitis”; “*Helicobacter pylori* AND periodontitis”; “*Helicobacter pylori* AND dental plaque”, to identify articles up to September 2021. The Newcastle-Ottawa scale was used to assess study quality. A meta-analysis was performed using RevMan 2020 (Cochane Collaboration) software. A total of 1,315 studies were identified and 12 were included, analyzing 226,086 patients with mean age between 10.5 and 63.4 years. The prevalence of *H. pylori* in the oral cavity ranged from 5.4 to 83.3%. A random-effects model was used to analyze the presence of *H. pylori* and subgroups were made according to the method of evaluation (PCR or RUT). Statistical significance was found in the overall analysis (*p* = 0.01). There is no clear evidence that *H. pylori* present in oral bacterial plaque causes gastric infection and vice versa.

**Systematic Review Registration:**

www.INPLASY.COM, identifier: INPLASY2021100097.

## Introduction

*Helicobacter pylori* (*H. pylori*) is a gram-negative bacterium capable of producing one of the most common gastric bacterial infections in humans, affecting about half of the world's population and is the main cause of chronic gastritis, gastroduodenal ulcer, and adenocarcinoma of the stomach (1, 2). Despite treatment by systemic antibiotic therapy, certain patients have persistence of infection after treatment (3). Therefore, some researchers have suggested that both dental plaque and saliva could act as a reservoir and have implications for reinfection once the bacterium is eradicated from the gastric tract; some studies have even considered that the mouth could be a source of constant reinfection and that eradication of the bacterium from the oral cavity would be more difficult than from the gastrointestinal area (4, 5).

Similarly, periodontal diseases (PDs), are multifactorial infectious diseases, associated with a microbiota composed predominantly of gram-negative species, which show a close relationship with many systemic diseases and affect a high percentage of the elderly human population, presenting irreversible destruction of the supporting structures of the teeth (6, 7). The association between PDs and certain systemic diseases is explained by inflammation or immune response to periodontal pathogens (8). In recent years, using techniques such as polymerase chain reaction (PCR), DNA sequencing and hybridization, about 1,000 bacterial species have been found in the oral cavity (9, 10).

In 1996, 11 pathogens associated with periodontitis (P) were identified, of which three were strongly associated and eight were moderately associated (11). The initiation and spread of PDs is due to a dysbiosis of the oral commensal microbiota (dental plaque). However, periodontitis is considered a complex disease, with etiological factors acting at numerous levels: microbial, host, environmental and genetic, which may predispose or protect against the disease (12–14).

The association between PDs and gastro-duodenal ulcer has only been investigated in a limited number of studies, which have shown a significant association between both pathologies, even suggesting that patients with PDs, who harbor *H. pylori* in the oral cavity, would be subsidiary to suffer from gastric pathologies (15–18).

Therefore, the aim of this systematic review and meta-analysis was to identify and analyze clinical studies to determine the direct correlation between PDs and *H. pylori* gastric infection.

## Materials and Methods

This systematic review and meta-analysis were reported using the Preferred Reporting Items for Systematic Reviews and Meta-Analyses (PRISMA) statement (19). INSPLAY registration number: INPLASY2021100097. The PRISMA 2009 Checklist is reported in Supplementary Material (Supplementary Table 1).

The research question was elaborated using the PICOS strategy: Is there a correlation between PDs and *H. pylori* gastric infection? The population (P) was defined as subjects with PD; the intervention (I) was defined as the diagnosis of PD; the comparison group (C) corresponded to subjects without PD; the outcome (O) was defined as *H. pylori* gastric infection; and the study design (S) was defined to include non-interventional, cross-sectional, or case-control cohort studies. This review had no year restrictions.

### Data Sources and Search Strategy

The PubMed, EMBASE and Web of Science (WOS) databases were searched to identify articles that were eligible until September 2021. The search terms used were: [MeSH terms] “*Helicobacter pylori* AND periodontal diseases,” [MeSH terms] “*Helicobacter pylori* AND gingivitis,” [MeSH terms] “*Helicobacter pylori* AND chronic periodontitis,” [MeSH terms] “*Helicobacter pylori* AND periodontitis” and [MeSH terms] “*Helicobacter pylori* AND dental plaque.” The Boolean operator “AND” was used to combine the searches. In addition, other articles were retrieved by hand searching recent reviews.

### Data Extraction and Quality Assessment

Two authors independently read the titles and abstracts of the articles. The bibliographic references of each study were also reviewed as possible sources to identify additional studies. When there was any discrepancy between the two authors, a third author was consulted to establish a consensus.

The Newcastle-Ottawa Scale (NOS) (20) was applied to assess the quality of all studies. The NOS checklist contains three quality parameters: (i) selected population, (ii) comparability of groups, and (iii) assessment of exposure or outcome of interest for case-control or cohort studies. Each study was assigned a score from 0 to 9. Studies with a score ≥7 were considered high quality articles. Discrepancies in quality assessment were discussed and resolved by two authors (NL-V and AL-V). Any discrepancies were resolved by discussion with a third investigator. Studies with scores of nine to seven stars were considered to be of high quality, four to six stars of moderate quality, and one to three stars of poor quality and at high risk of bias.

### Inclusion and Exclusion Criteria

Inclusion criteria were as follows:

(a) Clinical studies that provided data on *H. pylori* infection in both the stomach and oral cavity, confirmed by polymerase chain reaction (PCR) or rapid urease test (RUT).
(b) Clinical studies that associated PDs with *H. pylori*. The diagnosis of PD was confirmed according to the diagnostic criteria in periodontology.
(c) Types of studies: cross-sectional studies, cohort studies, and case-control studies.
(d) Studies published in English.

Exclusion criteria were as follows:

(a) Studies that did not provide association data (*H. pylori*/PDs gastric infection).
(b) Studies that correlated *H. pylori* with oral pathologies other than PDs.
(c) Informative studies, clinical cases or studies published in languages other than English.

### Statistical Analysis and Data Synthesis

The meta-analysis was performed with RevMan software (Review Manager (RevMan) [Software]. Version 5.4.1, The Cochrane Collaboration, 2020).

A meta-analysis based on Odds Ratio (OR) with 95% confidence intervals (CI) was performed for adverse event outcomes. Mean difference (MD) and standard deviation (SD) were used to estimate effect size. The random-effects model was selected because of the expected methodological heterogeneity in the included studies; furthermore, heterogeneity was interpreted as significant when the *I*^2^ value was >50% (21). The threshold for statistical significance was defined as *p* < 0.05. A funnel plot was used to assess publication bias.

Two subgroups were performed: one for studies using PCR as a diagnostic test for *H. pylori* and one for studies using RUT.

## Results

### Characteristics of Eligible Studies

**Figure 1:**
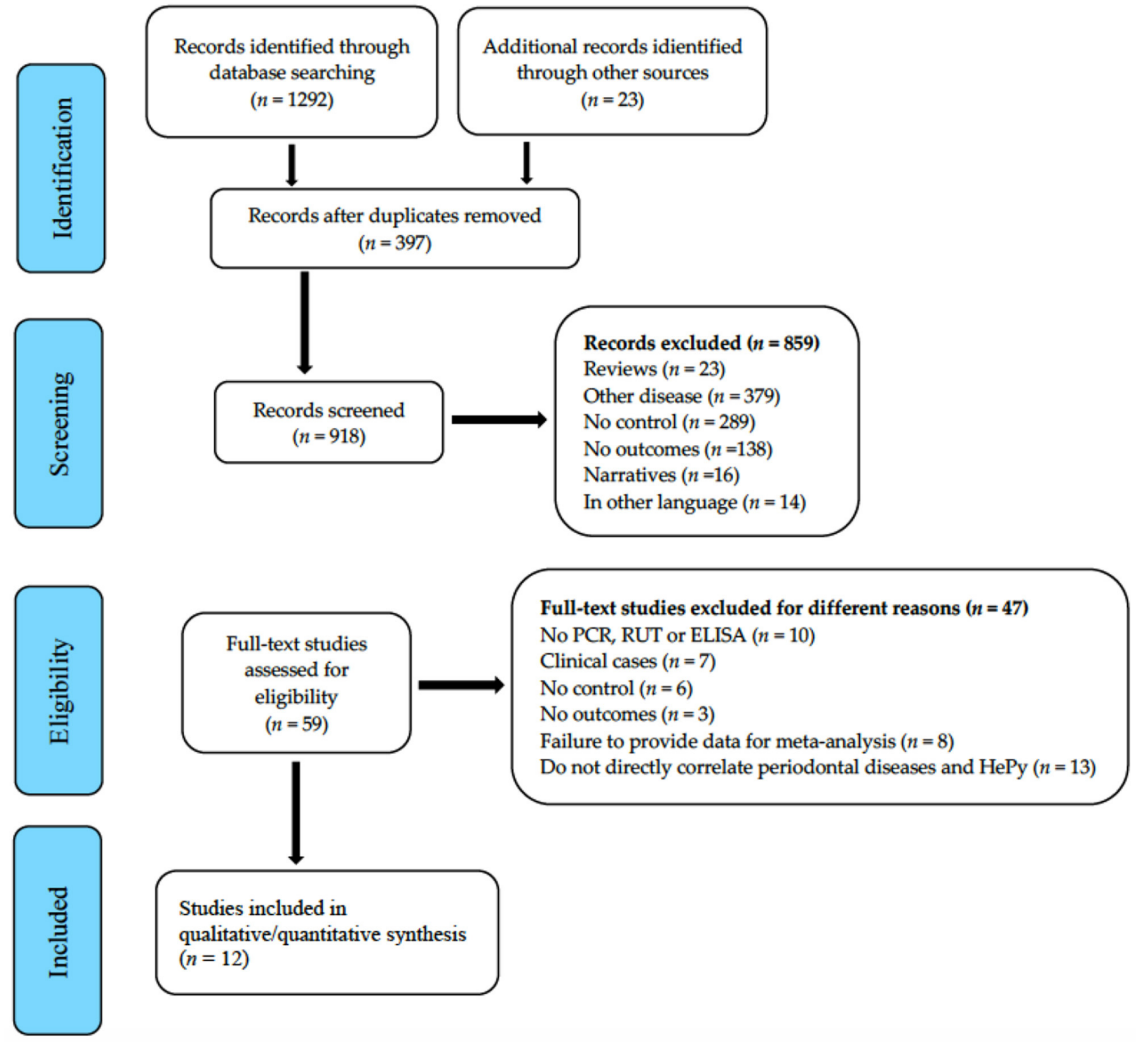
Flowchart.

A total of 1,315 articles were identified up to September 2021. Of the 1,315 studies searched, 1,256 (95.5%) papers were excluded by reviewing the abstract and title. After exclusion, a total of 12 eligible articles were included for analysis (22–33). Figure 1 (Flowchart) shows the article selection procedure. Eight of the selected studies were case-control (22–25, 27, 29, 30, 32) and four were cross-sectional studies (26, 28, 31, 33). Seven were conducted in the university setting (22, 23, 25, 28, 30, 31, 33) and six in hospital settings (24, 26, 27, 29, 32). The sample size ranged from 56 to 173,209 subjects; a total of 226,086 subjects were studied, with a mean age between 10.5 and 63.4 years. The prevalence of *H. pylori* in the oral cavity ranged from 5.4 to 83.3%. The characteristics of the included studies are shown in Tables 1, 2.

**Table 1:** Characteristics of studies.

**References**	**Sample size**	**Mean age (range years)**	**Design of study**	**Country**	**Source**
Teoman et al. (22)	67	Women: 41.03 ± 13.68 Men: 39.74 ± 12.05	Case-control	Turkey	University
Bürgers et al. (23)	94	53	Case-control	EE. UU.	University
Al Asqah et al. (24)	101	40.77 ± 14.15	Case-control	Saudi Arabia	Hospital
Eskandari et al. (25)	67	42.3 ± 12.52	Case-control	Iran	University
Boylan et al. (26)	51,529	65	Cross-sectional	EE. UU.	Hospital
Bharath et al. (27)	56	NR	Case-control	India	Hospital
Nisha et al. (28)	500	43.678	Cross-sectional	India	University
Yang et al. (29)	212	60	Case-control	China	Hospital
Aksit et al. (30)	100	10.5	Case-control	Turkey	University
Zahedi et al. (31)	86	40.32	Cross-sectional	Iran	University
Luo et al. (32)	65	47.5 ± 10.5	Case-control	China	Hospital
Byun et al. (33)	173,209	54.8 ± 7.9	Cross-sectional	Korea	University

*NR, not report*.

**Table 2:** *Helicobacter pylori* identification method and prevalence in oral cavity/plaque.

**References**	**Oral identification method**	**Gastric identification method**	**Prevalence of *H. pylori* in oral cavity**
Teoman et al. (22)	Dental plaque. PCR	Gastric biopsy	25.4%
Bürgers et al. (23)	Dental plaque. PCR	Gastric biopsy	5.4%
Al Asqah et al. (24)	Subgingival plaque. RUT	NR	49%
Eskandari et al. (25)	Dental plaque. PCR	Gastric biopsy	5.97%
Boylan et al. (26)	NR	NR	NR
Bharath et al. (27)	Dental plaque. PCR	Gastric biopsy	33.9%
Nisha et al. (28)	RUT	NR	61.4%
Yang et al. (29)	RUT	NR	NR
Aksit et al. (30)	PCR	Gastric biopsy	83.3%
Zahedi et al. (31)	NR	Gastric biopsy	NR
Luo et al. (32)	PCR	Endoscopy	NR
Byun et al. (33)	NR	NR	NR

*PCR, Polymerase Chain Reaction; RUT, Rapid Urease Test; NR, not report*.

### Quality of the Included Studies

Using the Newcastle-Ottawa scale, each study was assigned a score from 0 to 9. All studies scored ≥7 and were therefore of high quality. The highest score (9) was obtained by six of the twelve included studies (21, 22, 24, 28, 30, 31) and the lowest (7) by Boylan et al. (25) and Byun et al. (32) (Table 3).

**Table 3:** Assessment of the quality of included studies using the Newcastle-Ottawa Scale.

**References**	**Selection**	**Compatibility**	**Exposure/Result**	**Total, score**
Teoman et al. (22)	****	**	***	9
Bürgers et al. (23)	****	***	**	9
Al Asqah et al. (24)	****	***	*	8
Eskandari et al. (25)	****	***	**	9
Boylan et al. (26)	****	*	**	7
Bharath et al. (27)	****	***	*	8
Nisha et al. (28)	****	*	***	8
Yang et al. (29)	****	***	**	9
Aksit et al. (30)	****	**	**	8
Zahedi et al. (31)	****	***	**	9
Luo et al. (32)	****	**	***	9
Byun et al. (33)	****	*	**	7

### Quantitative Synthesis (Meta-Analysis Results)

**Figure 2:**
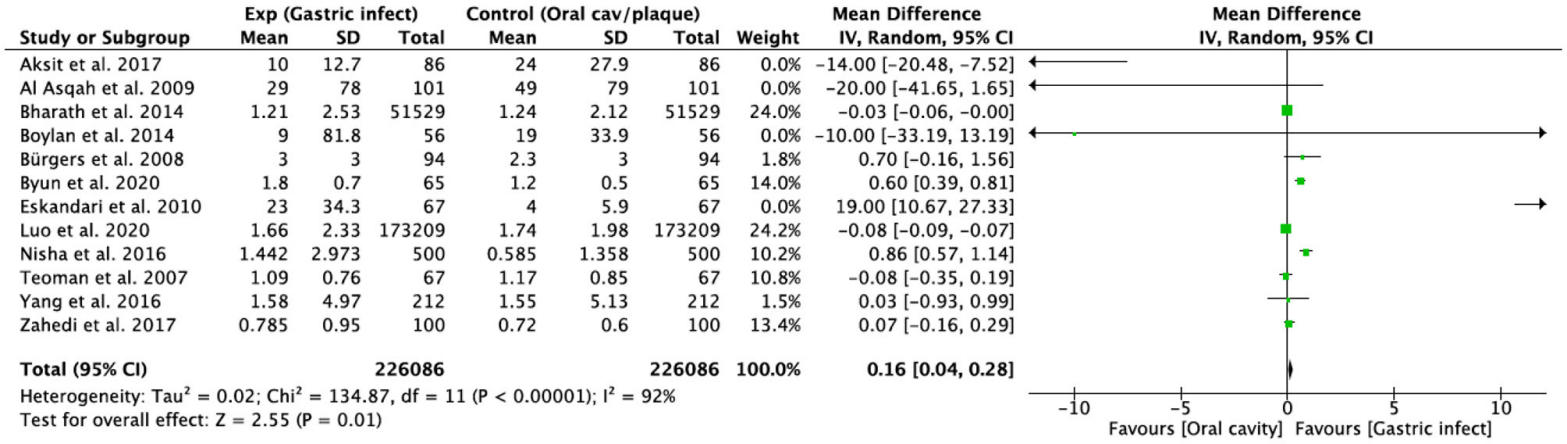
Forest plot of the prevalence of *H. pylori* gastric infection and oral cavity. SD, Standard Deviation; CI, Confidence Interval.

The same studies included in the qualitative synthesis were used to perform a meta-analysis comparing gastric *H. pylori* infection with the presence of the bacterium in the oral cavity. A forest plot was used to test the results. A meta-analysis of adverse outcomes could not be performed due to lack of data. Heterogeneity was very high (*I*^2^ = 92%), *p* = 0.01 indicating that patients with *H. pylori* gastric infection were positively associated with the presence of the bacteria in the oral cavity. The studies by Bharath et al. (27) and Luo et al. (32) had the highest weight (24 and 24.2%, respectively), due to sample size. The studies with the lowest weight were those of Al Asqah et al., Eskandari et al., Boylan et al., and Aksit et al. (24–26, 30) (Figure 2).

**Figure 3:**
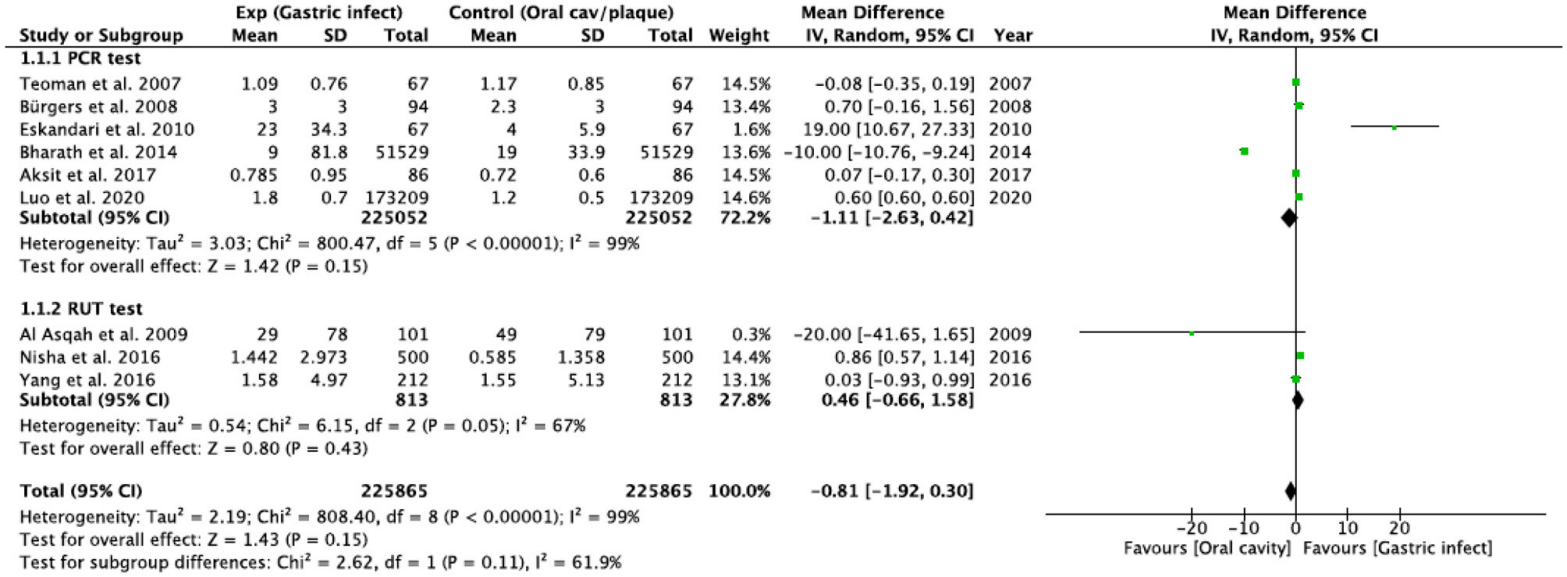
Subgroup analysis.

The subgroup analysis (studies that used PCR and studies that used RUT), presented greater heterogeneity (*I*^2^ = 99%) and no statistical significance was found (*p* = 0.11 for RUT and *p* = 0.15 for PCR) (Figure 3).

### Publication Bias and Heterogeneity

**Figure 4:**
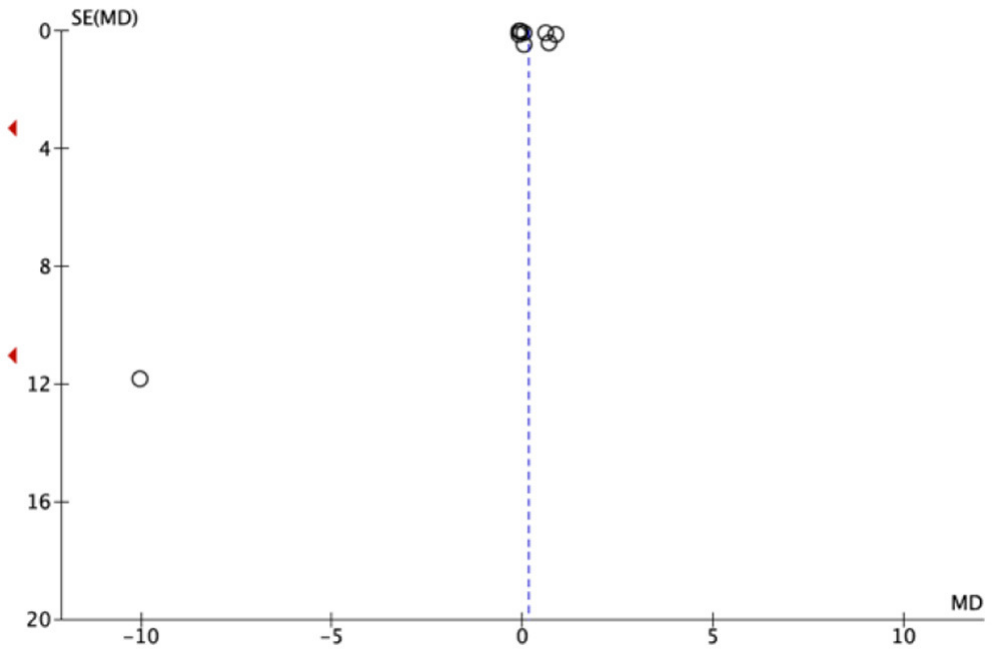
Funnel plot of all included studies.

**Figure 5:**
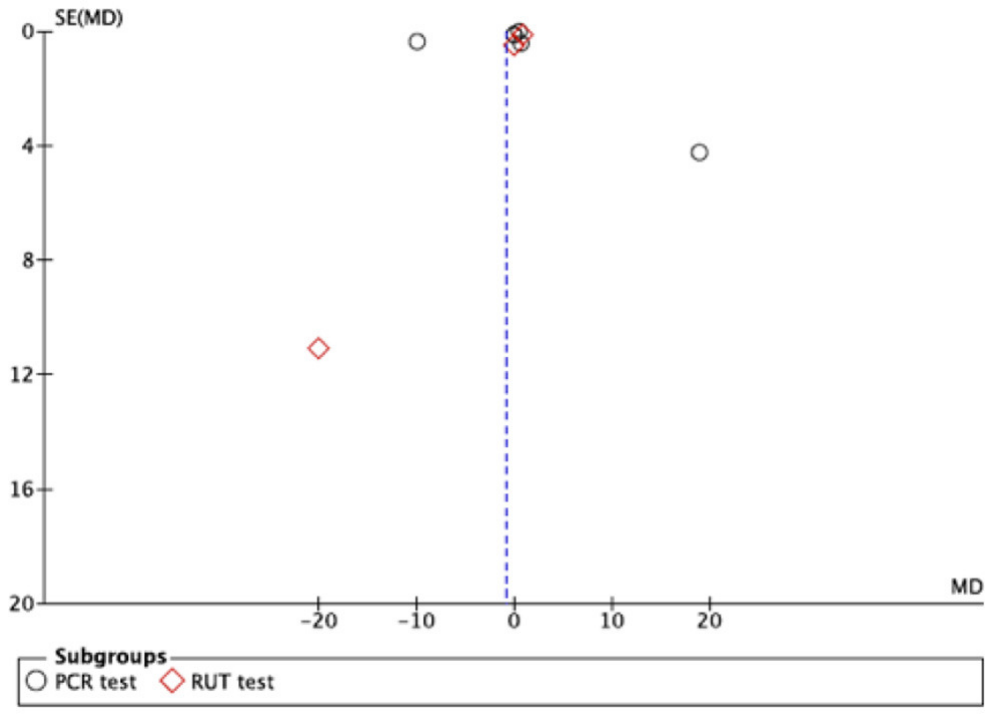
Funnel plot of subgroups.

The included studies showed important graphic signs of publication bias as can be seen in both Funnel Plots (Figures 4, 5).

## Discussion

The aim of the present study was to answer the following question: To what extent are periodontal diseases capable of producing gastric infection by *H. pylori*? To quantify the potential effect of this ability, a meta-analysis of studies that assessed the bacteria in dental plaque was performed.

*Helicobacter Pylori* is a common bacterium that colonizes the gastric mucosa and is also considered by many researchers to be a risk factor for certain oral diseases, such as PDs, canker sores, squamous cell carcinoma, burning tongue and halitosis (34).

Both the oral cavity and the peridontal pockets have been hypothetically considered as one of the mechanisms of reinfection and recolonization of *H. pylori* and that, conversely, the chronically infected condition of PD could favor the colonization of periodontal pockets by *H. pylori* (35, 36). In turn, certain studies have pointed to the oral cavity as the main colonizer of *H. pylori* and subsequent gastric infection indicating that there is a relationship between the presence of *H. pylori* in the oral cavity and the severity of periodontal disease (37–39). There are serious discrepancies among researchers on the presence of *H. pylori* in the oral cavity. Doré-Davin et al. (40) reported figures of 41% for the presence of *H. pylori* in the oral cavity; other studies have even reported 97% positivity for *H. pylori* in dental plaque samples (41), on the contrary, some studies even associate *H. pylori* infection with a lower risk of PD (42) arguing a lower *H. pylori* infection in patients with severe atrophic gastritis or differences between men and women, as female hormonal fluctuations due to pregnancy or menopause affect periodontopathic bacterial flora, saliva volume and viscosity, and oral mucosal vascularization (43). In addition, during pregnancy, there is a neglect of oral hygiene, associated with frequent vomiting can lead to dental erosions and even periodontitis. All this together with a postmenopausal loss of bone density and dental loss (44, 45). Kadota et al. (46) found higher rates of *Porphyromonas gingivalis, Treponema denticola*, and *Prevotella intermedia* in *H. pylori*-positive subjects than in *H. pylori*-negative subjects, highlighting the incidence of *H. pylori* and many periodontopathic bacterial species increases with age and concluding that *H. pylori* can coexist with specific periodontopathic bacterial species, although the interactions between the two bacteria have not been demonstrated. Umeda et al. (15), in a study of 57 subjects, highlighted the importance of removing *H. pylori* from the mouth because of the danger of colonization in the stomach and the importance of paying special attention to patients with periodontitis who harbor *H. pylori* in the oral cavity. In this regard, some authors have suggested that *H. pylori* recolonized in the gastric mucosa from dental plaque would be resistant to systemic antimicrobial therapy (47). A retrospective case-control study on a large population-based sample (177,240 patients), with a prolonged follow-up period (13 years), concluded a positive association between gastroduodenal ulceration and PD (48).

A review by Anand et al. (34) on the prevalence of *H. pylori* in dental plaque ranged from 0 to 100%, highlighting that the wide variation in the results could be explained by several factors, such as the characteristics of the sample population, the different sampling procedures and the different methodologies used to detect the microorganism in dental plaque; in our meta-analysis, these data ranged from 5.4 to 83.3%, due to the same factors.

On the other hand, due to the biofilm properties of dental plaque, it offers resistance to systemic antimicrobial treatments, thus *H. pylori* is not susceptible to synthetically administered antimicrobial agents and periodontal therapies that remove microbial deposits from dental plaque, including *H. pylori*, have been proposed in this regard (49, 50).

The methods for detecting dental plaque are another source of controversy among the authors, giving rise to great heterogeneity of the studies considered. In our meta-analysis we have included studies that identified *H. pylori* by PCR (21, 22, 24, 26, 29) and RUT methods (23, 27, 28) and others that did not report method of identification (25, 30, 32). Certain authors have criticized the reliability of RUT for detecting *H. pylori* in dental plaque, because *Streptococcus, Haemophilus*, and *Actinomyces* species can be detected as part of the normal oral flora, despite the fact that *H. pylori* is the only known urease-positive microorganism resident in the stomach (51). A study in 88 patients, published by Chitsazi et al. (52) on the prevalence of *H. pylori* in dental plaque, using RUT, reported a prevalence of <40%, questioning the validity of urease testing for diagnosing *H. pylori* in gastric infection; furthermore, the study authors found no association between *H. pylori* in dental plaque and gastric infection. Other studies, on the contrary, consider the RUT as the test with the best sensitivity (92.16%), although with a lower specificity than the PCR test (53).

On the other hand, reading urease tests before the recommended time can falsify the results (54) and, in addition, in commercial kit designs, the density of bacteria present in the sample affects the diagnostic accuracy, requiring a minimum of 10,000 microorganisms for the RUT result to be reliable; other factors such as the presence of blood in the biopsies or contamination with formalin distort the results, decreasing the sensitivity of the RUT (55–59).

Unlike RUT, the PCR method is used not only for the detection of *H. pylori*, but also to characterize pathogenic genes and mutations associated with antimicrobial resistance (60). Today, many modifications of PCR technology have been developed to increase the sensibility of detection, even to increase sensitivity to 100% (61). In recent years, a new PCR system has been developed, which uses primer sets specific to 48 *H. pylori* strains in order to increase the diagnostic accuracy of PCR in the oral cavity; however, despite being a highly sensitive method, certain authors have drawn attention to its poor ability to detect small amounts of bacteria, depending on the specificity and sensitivity of the primer used, for example, primers related to bacterial urease activity often prove to be a confounding factor (62). Amiri et al. (63) described a highly specific and sensitive DNA amplification method for the detection of *H. pylori* in dental plaque samples, showing that it had a higher detection rate than PCR by 66.67 and 44%, respectively.

For all these reasons, there is great confusion among the scientific community and the evidence supporting the role of the oral cavity as a significant reservoir of *H. pylori* is inconclusive, and it is necessary to establish the reasons for such discrepancies.

Dowsett and Kowolik in a review article (51) questioned whether the reservoir of *H. pylori* in the oral cavity would be significant for subsequent gastric infection and that there should be a microenvironment capable of supporting *H. pylori* growth, such as pH, oxidation/reduction (redox) potential and nutrient availability adequate to maintain *H. pylori*.

Finallly, another factor to take into account would be the geographic distribution of both pathologies. Gastric infection can be acquired from environmental sources, but in most circumstances, it is probably acquired interpersonally, by the fecal-oral or oral-oral route, depending on the circumstances, although the former is likely to be the more important (64); in contrast, the transmission of periodontal diseases is unclear, with genetically distinct types of *Porphyromonas gingivalis* considered to be more associated with the disease than others, but further studies are needed to relate this to ethnic differences (65).

Our meta-analysis found a moderate statistical significance (*p* = 0.01), however, in the subgroup study, no statistical significance was found (*p* = 0.15). However, the “lack of statistical significance” does not point in a specific direction, since, in certain situations, the relevant effects are not “statistically significant,” since a given association, may not be clinically or epidemiologically relevant. Therefore, we consider, according to the studies consulted and evaluated in this meta-analysis, that there may be situations in which certain periodontal situations can influence *H. pylori* gastric infection and vice versa.

Nevertheless, this study had several limitations: the small number of studies included in the meta-analysis; the included studies analyzed the presence of *H. pylori*, both in the bacterial plaque and in the gastric mucosa, in different territories and different assessment methods were used; finally, some studies did not report data.

For all these reasons, we consider that the results obtained should be taken with caution.

## Conclusions

The amount of *H. pylori* bacteria necessary to induce infection in the stomach is still unknown, as well as whether the presence of *H. pylori* in the mouth is transient and whether there are risk factors that favor its growth in the oral cavity.

Therefore, we believe that studies with well-designed and appropriate methodologies are needed to demonstrate a direct correlation between PDs and *H. pylori* gastric infection.

## Data Availability Statement

The original contributions presented in the study are included in the article/Supplementary Material, further inquiries can be directed to the corresponding author/s.

## Author Contributions

NL-V and AL-V: conceptualization and writing—review and editing. JMA and BM: methodology. AL-V, CR, and AS: validation. NL-V: formal analysis. NL-V and BM: data curation. AL-V: supervision. All authors have read and agreed to the published version of the manuscript. All authors contributed to the article and approved the submitted version.

## Conflict of Interest

The authors declare that the research was conducted in the absence of any commercial or financial relationships that could be construed as a potential conflict of interest.

## Publisher's Note

All claims expressed in this article are solely those of the authors and do not necessarily represent those of their affiliated organizations, or those of the publisher, the editors and the reviewers. Any product that may be evaluated in this article, or claim that may be made by its manufacturer, is not guaranteed or endorsed by the publisher.
